# A Five-Year Review of Newborn Screening for Spinal Muscular Atrophy in the State of Utah: Lessons Learned

**DOI:** 10.3390/ijns10030054

**Published:** 2024-07-22

**Authors:** Kristen N. Wong, Melissa McIntyre, Sabina Cook, Kim Hart, Amelia Wilson, Sarah Moldt, Andreas Rohrwasser, Russell J. Butterfield

**Affiliations:** 1 Department of Pediatrics, University of Utah, Salt Lake City, UT 84113, USA; 2 Utah Newborn Screening Program, Salt Lake City, UT 84129, USA; 3 Myotonic Dystrophy Foundation, Oakland, CA 94612, USA

**Keywords:** spinal muscular atrophy, newborn screening, outcomes

## Abstract

Spinal muscular atrophy (SMA) is an autosomal recessive condition characterized by alpha motor neuron degeneration in the spinal cord anterior horn. Clinical symptoms manifest in the first weeks to months of life in the most severe cases, resulting in progressive symmetrical weakness and atrophy of the proximal voluntary muscles. Approximately 95% of SMA patients present with homozygous deletion of the *SMN1* gene. With multiple available therapies preventing symptom development and slowing disease progression, newborn screening for SMA is essential to identify at-risk individuals. From 2018 to 2023, a total of 239,844 infants were screened. 13 positive screens were confirmed to have SMA. An additional case was determined to be a false positive. We are not aware of any false-negative cases. All patients were seen promptly, with diagnosis confirmed within 1 week of the initial clinical visit. Patients were treated with nusinersen or onasemnogene abeparvovec. Treated patients with two copies of *SMN2* are meeting important developmental milestones inconsistent with the natural history of type 1 SMA. Patients with 3–4 copies of *SMN2* follow normal developmental timelines. Newborn screening is an effective tool for the early identification and treatment of patients with SMA. Presymptomatic treatment dramatically shifts the natural history of SMA, with most patients meeting appropriate developmental milestones. Patients with two copies of *SMN2* identified through newborn screening constitute a neurogenetic emergency. Due to the complexities of follow-up, a multidisciplinary team, including close communication with the newborn screening program, is required to facilitate timely diagnosis and treatment.

## 1. Introduction

Spinal muscular atrophy (SMA) is an autosomal recessive neuromuscular condition caused by pathogenic variants in the *SMN1* gene which leads to the degeneration and loss of alpha motor neurons in the anterior horn of the spinal cord. Approximately 95% of cases are due to a homozygous deletion of exon 7 in *SMN1,* with the remaining 5% of cases due to compound heterozygous variants in *SMN1*, typically a deletion of *SMN1* and a pathogenic sequence variant. The *SMN2* gene is a pseudogene of *SMN1* located proximally to *SMN1* on chromosome 5q. Due to a single base pair transition in exon 7 disrupting a splice enhancer, the majority of *SMN2* transcripts exclude exon 7 and produce a non-functional SMN protein that is quickly degraded. Differential splicing results in 10% of transcripts including exon 7, thereby generating a functional SMN protein. Thus, *SMN2* modifies the age of onset and severity of SMA, with higher copy numbers of *SMN2* leading to milder and later onset presentations due to increased residual levels of functional SMN protein [1].

Clinically, SMA presents with progressive symmetric muscle weakness and atrophy of the proximal voluntary muscles and resulting motor impairments. Historically, patients with SMA were assigned a clinical type based on the highest motor milestone achievement. Age of onset can range from the first few weeks of life to adulthood [1,2,3]. The most common type of SMA is type 1, which presents within the first 6 months of life and is defined by the inability to sit independently [3]. Patients with type 1 SMA most commonly present with two copies of *SMN2* [4]. In these severe cases, clinical symptoms can be identified in the first weeks to months of life [5]. There has been rapid development of multiple disease-modifying therapies available to prevent symptom development or slow disease progression. These include *SMN2* modulators such as nusinersen (NU) and risdiplam as well as the *SMN1* gene replacement therapy onasemnogene abeparvovec (OA) [6,7,8,9,10,11]. Emerging data show dramatically improved outcomes with early treatment before the onset of symptoms [6,7,8]. With these new therapies, the importance of early treatment to maximize motor outcomes in patients was increasingly considered and SMA soon met the accepted criteria for newborn screening (NBS) [12,13]. In January 2018, Utah became the first state in the United States to perform population-wide state newborn screening for SMA, and SMA was ultimately added to the Recommended Uniform Screening Panel by the Advisory Committee on Heritable Disorders in Newborns and Children later that year. Thus, newborn screening for SMA has been essential in identifying individuals who can benefit from early treatment initiation [14,15,16]. As of January 2024, all newborns in the U.S. are screened for SMA [17].

This retrospective study reviews the experience of SMA newborn screening and subsequent clinical follow-up for the first five years in the state of Utah.

## 2. Materials and Methods

A retrospective review of newborn screening results and clinical outcomes in the state of Utah from 28 January 2018 to 27 January 2023 was completed. This study was completed under a University of Utah Institutional Review Board-approved protocol.

The newborn screening assay consists of quantitative polymerase chain reaction (PCR) to detect the presence or absence of exon 7 in the *SMN1* gene (Figure S1). Utah is a two-screen state, testing newborns typically 24–48 h after birth and then again at approximately two weeks of age. All first specimens received are tested for SMA. Utah uses a laboratory-developed test (LDT) to screen for both SMA and severe combined immunodeficiency (SCID) simultaneously. The LDT is a real-time triplex PCR assay in which three targets are measured concurrently: (1) *SMN1* for the detection of SMA, (2) T-cell receptor excision circles (*TREC*) for the detection of SCID, and (3) *RPPH1*, a housekeeping gene that is used as an internal control for the assay. A DNA isolate is prepared from a 3.2 mm dried blood spot punch. PCR Master Mix containing forward, reverse, and allele-specific probes for the three target amplicons is added. Samples are transferred to a Roche LightCycler II PCR instrument where fluorescence is measured in real time over 45 PCR cycles. Neither compound heterozygous patients who have a single deletion of *SMN1* and a second allele with a sequence variant nor heterozygous carriers of *SMN1* deletion are detected by this assay.

The results are integrated into a laboratory information system (LIMS) and are categorized as normal, abnormal, or indeterminate. Testing is repeated for samples that do not amplify prior to a result designation of abnormal or indeterminate. An abnormal result indicates that there is no amplification of *SMN1* and sufficient amplification of *RPPH1*. An indeterminate result indicates that there is late or poor amplification of *SMN1* and *RPPH1* (Table S1).

If a first newborn screen sample is abnormal for SMA, the assay will be urgently repeated for confirmation prior to reporting. Once confirmed, the NBS genetic counselors contact the primary care provider and neurology follow-up team who then coordinate an initial clinical visit. This initial visit includes a detailed neurologic examination, confirmatory diagnostic testing of *SMN1* and *SMN2*, anti-Adeno-Associated Virus 9(AAV9) antibody testing (if present, this can delay the delivery of *SMN1* gene replacement therapy until the resolution of antibodies which are assumed to be transplacental in origin), and discussion of treatment options.

Data gathered for review included critical time points in our SMA NBS workflow, the timing of clinical follow-up, confirmatory genetic test results, and treatment type and timing. Descriptive statistics were derived using Microsoft Excel 16. Clinical outcomes were monitored with longitudinal measurements via the Children’s Hospital of Philadelphia Infant Test of Neuromuscular Disorders (CHOP INTEND) and the gross motor subtest of the Bayley Scales of Infant and Toddler Development third edition (BSID) administered by the neuromuscular physical therapy team. The CHOP INTEND is an SMA-specific scale designed to assess motor function in very weak SMA infants, with higher scores representing better motor function [18,19]. The BSID is a norm-referenced developmental test focused on skill acquisition for infants 16 days to 42 months of age [20]. Parental and physician reports of developmental milestones were also obtained from chart reviews and concatenated with these objective measures to determine overall developmental milestone tracking for each patient.

## 3. Results

In our five-year study period, a total of 239,844 infants were screened for SMA in Utah with 14 screen-positives (Table 1).

One of the fourteen screen-positive cases was a false positive. This was the first positive screen called out after starting screening in Utah. On review of the case, the initial run of this sample showed no amplification of the control housekeeping gene, *RPPH1*. Upon repeat run, no amplification of *SMN1* was identified and late amplification of *RPPH1* was observed. Given the concern for absent *SMN1* amplification, this was called out to the clinical team as a positive screen despite concerns that it could be a false positive. Clinical confirmatory testing of *SMN1* and *SMN2* in that case revealed two copies of *SMN1* and one copy of *SMN2* in the patient, consistent with them being unaffected by SMA. A repeat second NBS screen showed normal amplification of *SMN1*. Thus, the first abnormal but false positive screen was thought to be due to poor sample quality. To date, we are not aware of any false negative screens over the first five years of screening.

**Figure 1:**
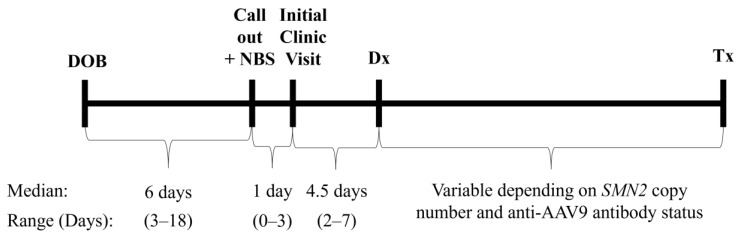
Critical time points in SMA NBS follow-up. One case was excluded as confirmatory testing was sent prior to the return of positive NBS due to a known prenatal diagnosis. DOB, date of birth; NBS, newborn screen; Dx, diagnosis; Tx, treatment.

The time to notification of the neurology team for a positive screen was a median of 6 days (range 3–18 days) from the date of birth. Of note, there was one outlier with late screening due to home birth, which resulted in the first newborn screening sample for this case being sent at the 2-week well-child check. The median time from the collection of newborn screen samples to call out of the positive screen was 5 days. The time to the first clinical visit was typically 1 day. Confirmatory *SMN1* and *SMN2* diagnostic genetic testing as well as anti-AAV9 antibody testing were sent at the initial clinic visit. The time to a confirmed positive clinical diagnostic genetic testing result was a median of 4.5 days from the initial clinic visit (Figure 1).

Thirteen positive screens were clinically confirmed to have SMA via diagnostic genetic testing with homozygous deletion of *SMN1* and variable *SMN2* copy numbers (Table 2). One of these cases was previously known to our programs due to prenatal diagnosis in another state; however, the patient had undergone a newborn screen locally after undergoing adoption in Utah. Excluding this out-of-state adoption case, the incidence of SMA in Utah during our 5-year period was approximately 1 in 20,000 (12/239,884) live births.

Treatment modality and timing varied based on *SMN2* copy number and anti-AAV9 antibody status. Two patients were treated in clinical trials with gene replacement therapy. One patient with one copy of *SMN2* was severely affected at birth with their clinical presentation consistent with the prenatal onset of SMA type 0. In this case, the family opted for palliative care. Two patients with two copies of *SMN2* were positive for anti-AAV9 antibodies and were initially treated with nusinersen as a bridge to OA. Both of these patients received four loading doses of nusinersen and later received OA once antibody levels dropped. All remaining patients received OA as first-line treatment. Time to treatment for patients with two copies of *SMN2* was a median of 13.5 days from the initial clinical visit. Time to treatment for patients with three copies and four copies of *SMN2* was a median of 39 days and 89 days, respectively (Table 2).

Four out of the thirteen (31%) confirmed cases of SMA had elevated anti-AAV9 antibodies upon initial screening. Three of these patients underwent monthly routine anti-AAV9 antibody screening for the resolution of elevated antibodies. Anti-AAV9 antibodies resolved between two and four months for these patients. One of these patients presented as type 0 SMA and the family opted not to treat; thus, follow-up anti-AAV9 antibody testing was not completed in that case (Table 3).

**Figure 2:**
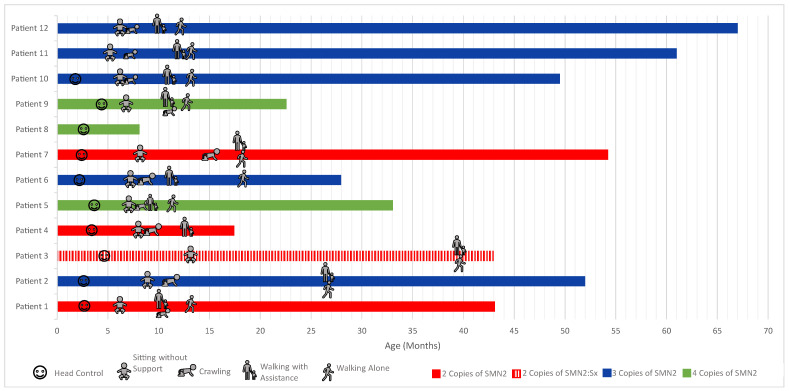
Observed attainment of developmental milestones in the treated patients. Developmental milestones were observed as obtained by the above time as assessed and/or recorded during routine physical therapy/neurology follow-up or by parent report. The end of the bars represents the current patient age. One patient with SMA type 0 clinical presentation was excluded from this figure as the patient did not meet any developmental milestones.

**Figure 3:**
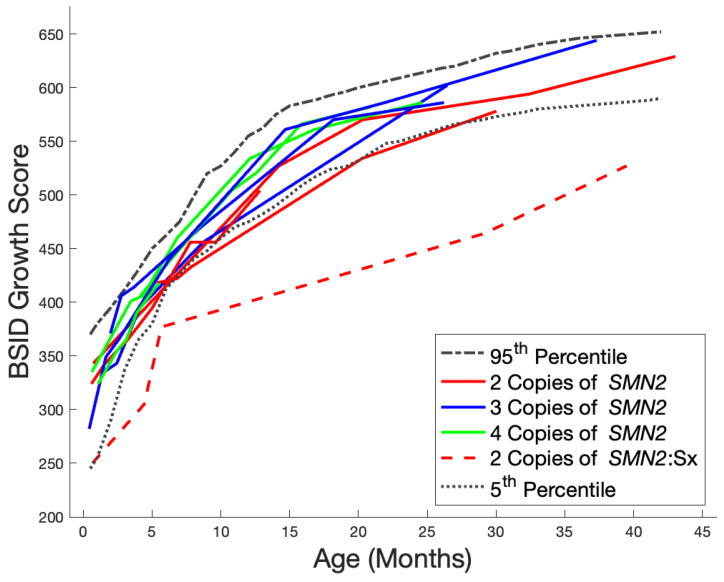
Longitudinal developmental outcomes (BSID Gross Motor Growth Scores). The color of the lines corresponds to the *SMN2* copy number of the patient (red = 2 *SMN2* copies, blue = 3 *SMN2* copies, and green = 4 *SMN2* copies); solid lines represent asymptomatic patients at the time of treatment; dashed lines represent symptomatic patients at the time of treatment; the dashed black line represents the 95th percentile of neurotypical age-matched peers; and the dotted black line represents the 5th percentile of neurotypical age-matched peers. Two patients were excluded from analysis due to treatment in clinical trials; thus, detailed developmental outcomes were unavailable. However, both are noted to be typically developing. A third patient with SMA type 0 clinical presentation was also excluded due to a BSID score of 0.

Developmental outcomes were measured with the CHOP INTEND, BSID, and parental and physician reports of gross motor developmental milestones during the 5-year study period. Treated patients with two copies of *SMN2* met early developmental milestones inconsistent with the natural history of SMA. Treated patients with three or four copies of *SMN2* are following normal developmental timelines (Figure 2 and Figure 3).

The majority of the treated NBS-identified patients demonstrated gross motor development between the 5th and 95th percentiles as compared to neurotypical age-matched peers as measured using BSID growth scores (Figure 3). In the first months of monitoring, patients with two copies of *SMN2* appear to fall within the middle of these percentiles. These two-copy *SMN2* patients then tend to fall toward the lower percentiles, while patients with three or four copies of *SMN2* continue within the middle percentiles over their developmental trajectory. All but one patient was thought to be clinically asymptomatic at the time of treatment. The one patient who was symptomatic prior to treatment (patient 3 in Figure 2) was identified on day of life 5 with a positive newborn screen. Confirmatory diagnostic testing showed absent *SMN1* and two copies of *SMN2* consistent with predicted type 1 SMA. This patient was also found to have elevated anti-AAV9 antibodies. At a follow-up visit on day of life 12, the patient had a normal neurologic exam. Due to ongoing discussions with the patient’s insurance provider about authorization and coverage for nusinersen vs. nusinersen with a bridge to OA therapy, there was a few days’ delay in starting treatment. Within this week’s timeframe, the patient became symptomatic, presenting with hypotonia and diaphragmatic breathing. Treatment with nusinersen was started on day of life 20, and eventually, the patient was treated at four months with OA. Upon the initiation of nusinersen on day of life 20, this patient had significant gross motor delays, weakness, and dysphagia with aspiration but currently continues to make developmental progress and is taking independent steps.

**Figure 4:**
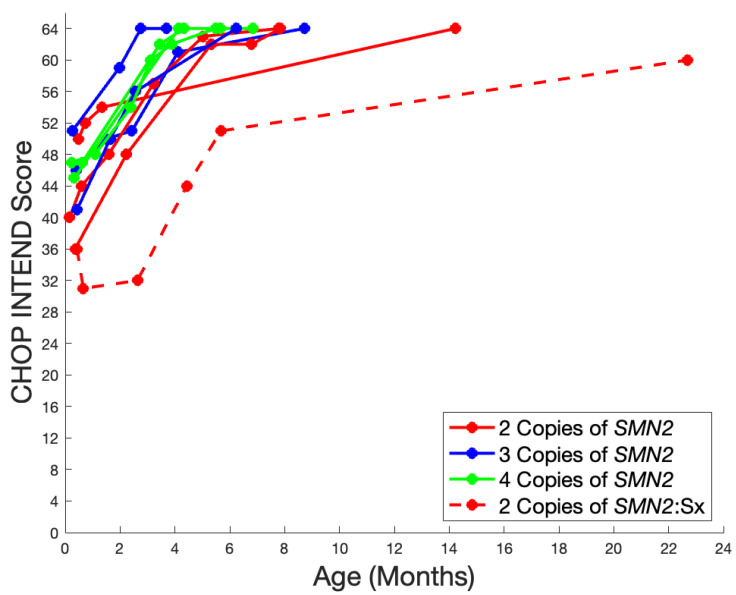
Longitudinal CHOP INTEND scores in the Utah NBS cohort. Visit time points are represented with circles and connected with lines for visualization purposes. The color of the lines corresponds to the *SMN2* copy number of a patient (red = 2 *SMN2* copies, blue = 3 *SMN2* copies, and green = 4 *SMN2* copies); solid lines represent asymptomatic patients at the time of treatment; and dashed lines represent symptomatic patients at the time of treatment. Two patients were excluded from analysis due to treatment in clinical trials; thus, detailed developmental outcomes were unavailable. However, both are noted to be typically developing. A third patient with SMA type 0 clinical presentation was also excluded.

CHOP INTEND scores in these infants ranged from 35 to 51 at initial evaluations; the majority of infants recorded a maximal score of 64 by 8 months of age. Note, CHOP INTEND scores were collected at routine clinical visits with variable time points, so for some infants, a delay in obtaining a maximal CHOP INTEND score was due to their follow-up schedule and not their motor function. The one infant referenced above who was symptomatic at the time of treatment achieved their highest score of 60/64 at 22.7 months of age (Figure 4).

## 4. Discussion

Utah was the first state in the United States to implement population-wide state newborn screening for SMA in January 2018. The incidence in our population during the first 5 years of screening was approximately 1 in 20,000 live births. This was surprisingly low compared to the typically quoted incidence of 1 in 10,000 births [1,3,21]. However, this is broadly consistent with updated published epidemiological data on SMA [5,14]. It is unclear what may be contributing to this lower incidence of SMA, although it is possible that additional years of data will be required before a definitive trend for incidence becomes apparent. Routine offering of carrier screening to all pregnant individuals in accordance with the American College of Obstetricians and Gynecologists recommendation may also be impacting the incidence of SMA as at-risk couples may be able to better assess their risk before or early in pregnancy [22]. It is also possible that as broader genetic testing has become available, patients who may have historically been given clinical diagnoses of SMA are now receiving more specific genetic diagnoses.

This study further expands the evidence base that newborn screening is an effective tool for the early identification and treatment of patients with SMA [14,23]. In terms of sensitivity, we are not aware of any false negative cases over the five years since starting screening. Since patients with type 1 or type 2 SMA are expected to become symptomatic within the first year of life and the state of Utah has a single pediatric neuromuscular center where all potential cases are referred, we are confident that we have not missed false negative cases in the community. However, we do acknowledge that there could be type 3 or 4 SMA cases that could be pre-symptomatic and unidentified at this time. Notably, an estimated 5% of cases are expected to be missed due to them being compound heterozygous for deletion in *SMN1* and a second pathogenic sequence variant [1,3]. SMA deletion/duplication and sequence analysis will continue to be a critical consideration for patients with clinical presentation concerning for SMA to identify compound heterozygous patients and other false negative screens.

Accurate measurement of an infant’s baseline motor capacity and subsequent motor development is essential to monitoring response to treatment. CHOP INTEND is a valid, clinically feasible, SMA-specific, and widely used motor scale for infants with SMA; however, it was designed for very weak infants with SMA type I, aged 1.4–260 months [18,19]. Infants diagnosed via NBS are much younger and primarily in a clinically silent or prodromal disease state at baseline assessment. In turn, early motor signs of SMA may not be reflected in their scores. A 2022 publication reported that baseline CHOP INTEND scores below 30 were associated with sub-optional outcomes, scores above 50 were associated with good prognosis, and scores 30–50 were not predictive of future motor outcomes [24]. The BSID provides a complementary assessment to the CHOP INTEND as it is designed to assess developmental status in early childhood [20]. However, this scale also has limitations in utility as it is designed to assess motor skill acquisition, and SMA natural history shows that there can be substantial loss of motor neurons and muscle strength before the regression of motor skills [25,26]. Emerging technologies such as wearable sensors have been proposed as a tool for the early identification of motor impairment and developmental delay [27,28,29]. These technologies may have particular application for prodromal infants with SMA identified via NBS and other neuromuscular disorders to aid in monitoring disease progression and treatment response in these youngest patients [30].

Prompt treatment before symptom onset resulted in a dramatic shift in the natural history of patients with SMA, with most of our NBS patients meeting appropriate developmental milestones. As illustrated in patient 3, timely diagnosis and treatment, particularly for patients with two copies of *SMN2*, can be critical as the difference of a few days may drastically impact developmental outcomes. Patients with 3 or 4 copies of *SMN2* were noted to have longer times to treatment for a variety of reasons including elevated anti-AAV9 antibodies requiring time to resolution, insurance issues, and less time pressure compared to patients with two *SMN2* copies. Updated treatment recommendations suggest that patients with two, three, or four copies of SMN2 should receive immediate treatment, and we aim to continue to mitigate delays for all patients with SMA and improve time efficiency in alignment with these recommendations [15,16]. With the approval of multiple therapies in the newborn period, continued re-evaluation of the treatment algorithm based on *SMN2* copy number and anti-AAV9 antibody status will be critical when considering bridged or combination therapies for the newborn screening patient population. Due to these complexities of newborn screening follow-up, a multidisciplinary team, including close communication with the newborn screening program, is required to facilitate diagnosis and treatment in a timely manner; thus, continued efforts to further expedite diagnostic testing, evaluation, and treatment are essential as the landscape of SMA diagnosis and treatment continue to evolve.

## Figures and Tables

**Table 1:** 5-Year summary of SMA NBS outcomes in Utah.

Year	Number of Screens	Number of Positive Screens
2018	48,218	3
2019	46,832	3
2020	46,862	3
2021	47,503	3
2022	46,754	2
To 27 January 2023	3675	0
Total	239,844	14

**Table 2:** Treatment and timing stratified by *SMN2* copy number.

*SMN2* Copy #	# of Cases	Treatment			Median Time to Treatment from Initial Clinic Visit in Days (Range)	Median Age at Treatment in Days (Range)
		Palliative	NU + OA	OA		
1	1 (8%)	1			-	-
2	4 (31%)		2	2	13.5 (7–16)	19 (15–23)
3	5 (38%)			5	39 (15–201)	47 (23–210)
4	3 (23%)			3	89 (87–182)	106 (96–187)

Abbreviations: NU, nusinersen; OA, onasemnogene abeparvovec; NU + OA, initial treatment with nusinersen with subsequent onasemnogene abeparvovec; #, number.

**Table 3:** Initial elevated anti-AAV9 antibody results and time to resolution.

Patient *SMN2* Copy Number	Anti-AAV9 Antibody Technical Result (Normal < 1:25)	Time to Normal Anti-AAV9 Antibody Result from Initial Abnormal (Months)
1	≥1:200	N/A
2	≥1:200	4
2	1:50	3.3
4	≥1:200	2.1

Four patients were identified to have elevated anti-AAV9 antibodies upon initial screening. One patient presenting as SMA type 0 did not undergo ongoing antibody screening as the family opted not to treat. N/A, not applicable.

## Data Availability

The data presented in this study are available on request from the corresponding author.

## Supplementary Materials

The following supporting information can be downloaded at: https://www.mdpi.com/article/10.3390/ijns10030054/s1, Figure S1: Schematic map indicating the location of qPCR primers used in the Utah newborn screening assay; Table S1: SMA Newborn Screening Results Interpretation and Follow Up.

## References

[1] Prior T.W., Leach M.E., Finanger E., Adam M.P., Feldman J., Mirzaa G.M., Pagon R.A., Wallace S.E., Bean L.J.H., Gripp K.W., Amemiya A. (2020). Spinal Muscular Atrophy. [2000 February 24 Updated 2020 December 3]. GeneReviews^®^ [Internet].

[2] Nicolau S., Waldrop M.A., Connolly A.M., Mendell J.R. (2021). Spinal Muscular Atrophy. Seminars in Pediatric Neurology.

[3] Nance J.R. (2020). Spinal Muscular Atrophy. Continuum Lifelong Learn. Neurol..

[4] Calucho M., Bernal S., Alías L., March F., Venceslá A., Rodríguez-Álvarez F.J., Aller E., Fernández R.M., Borrego S., Millán J.M. (2018). Correlation between SMA type and SMN2 copy number revisited: An analysis of 625 unrelated Spanish patients and a compilation of 2834 reported cases. Neuromuscul. Disord..

[5] Verhaart I.E., Robertson A., Wilson I.J., Aartsma-Rus A., Cameron S., Jones C.C., Cook S.F., Lochmüller H. (2017). Prevalence, incidence and carrier frequency of 5q-linked spinal muscular atrophy—A literature review. Orphanet J. Rare Dis..

[6] Strauss K.A., Farrar M.A., Muntoni F., Saito K., Mendell J.R., Servais L., McMillan H.J., Finkel R.S., Swoboda K.J., Kwon J.M. (2022). Onasemnogene abeparvovec for presymptomatic infants with three copies of SMN2 at risk for spinal muscular atrophy: The Phase III SPR1NT trial. Nat. Med..

[7] Strauss K.A., Farrar M.A., Muntoni F., Saito K., Mendell J.R., Servais L., McMillan H.J., Finkel R.S., Swoboda K.J., Kwon J.M. (2022). Onasemnogene abeparvovec for presymptomatic infants with two copies of SMN2 at risk for spinal muscular atrophy type 1: The Phase III SPR1NT trial. Nat. Med..

[8] Darryl C., Bertini E., Swoboda K.J., Hwu W.L., Crawford T.O., Finkel R.S., Kirschner J., Kuntz N.L., Parsons J.A., Ryan M.M. (2019). Nusinersen initiated in infants during the presymptomatic stage of spinal muscular atrophy: Interim efficacy and safety results from the Phase 2 NURTURE study. Neuromuscul. Disord..

[9] Crawford T.O., Swoboda K.J., De Vivo D.C., Bertini E., Hwu W.L., Finkel R.S., Kirschner J., Kuntz N.L., Nazario A.N., Parsons J.A. (2023). Continued benefit of nusinersen initiated in the presymptomatic stage of spinal muscular atrophy: 5-year update of the NURTURE study. Muscle Nerve.

[10] Mendell J.R., Al-Zaidy S., Shell R., Arnold W.D., Rodino-Klapac L.R., Prior T.W., Lowes L., Alfano L., Berry K., Church K. (2017). Single-Dose Gene-Replacement Therapy for Spinal Muscular Atrophy. N. Engl. J. Med..

[11] Chiriboga C.A., Swoboda K.J., Darras B.T., Iannaccone S.T., Montes J., De Vivo D.C., Norris D.A., Bennett C.F., Bishop K.M. (2016). Results from a phase 1 study of nusinersen (ISIS-SMN Rx) in children with spinal muscular atrophy. Neurology.

[12] Rousseau F., Gigure Y., Berthier M.-T., Gurette D., Girard J.-G., Dry M. (2012). Newborn Screening by Tandem Mass Spectrometry: Impacts, Implications and Perspectives. Tandem Mass Spectrometry—Applications and Principles.

[13] Watson M.S., Mann M.Y., Lloyd-Puryear M.A., Rinaldo P., Howell R.R. (2006). Newborn screening: Toward a uniform screening panel and system. Genet. Med..

[14] Lee B.H., Deng S., Chiriboga C.A., Kay D.M., Irumudomon O., Laureta E., Delfiner L., Treidler S.O., Anziska Y., Sakonju A. (2022). Newborn Screening for Spinal Muscular Atrophy in New York State: Clinical Outcomes From the First 3 Years. Neurology.

[15] Glascock J., Sampson J., Haidet-Phillips A., Connolly A., Darras B., Day J., Finkel R., Howell R.R., Klinger K., Kuntz N. (2018). Treatment Algorithm for Infants Diagnosed with Spinal Muscular Atrophy through Newborn Screening. J. Neuromuscul. Dis..

[16] Glascock J., Sampson J., Connolly A.M., Darras B.T., Day J.W., Finkel R., Howell R.R., Klinger K.W., Kuntz N., Prior T. (2020). Revised Recommendations for the Treatment of Infants Diagnosed with Spinal Muscular Atrophy Via Newborn Screening Who Have 4 Copies of SMN2. J. Neuromuscul. Dis..

[17] CureSMA 100% of States Now Screening Newborns for SMA. https://www.curesma.org/100-of-states-now-screening-newborns-for-sma/.

[18] Glanzman A.M., Mazzone E., Main M., Pelliccioni M., Wood J., Swoboda K.J., Scott C., Pane M., Messina S., Bertini E. (2010). The Children’s Hospital of Philadelphia Infant Test of Neuromuscular Disorders (CHOP INTEND): Test development and reliability. Neuromuscul. Disord..

[19] Glanzman A.M., McDermott M.P., Montes J., Martens W.B., Flickinger J., Riley S., Quigley J., Dunaway S., O’Hagen J., Deng L. (2011). Validation of the Children’s Hospital of Philadelphia Infant Test of Neuromuscular Disorders (CHOP INTEND). Pediatr. Phys. Ther..

[20] Bayley N. (2005). Bayley Scales of Infant and Toddler Development.

[21] Sugarman E.A., Nagan N., Zhu H., Akmaev V.R., Zhou Z., Rohlfs E.M., Flynn K., Hendrickson B.C., Scholl T., Sirko-Osadsa D.A. (2012). Pan-ethnic carrier screening and prenatal diagnosis for spinal muscular atrophy: Clinical laboratory analysis of >72,400 specimens. Eur. J. Hum. Genet..

[22] Romero S., Biggio J.R., Saller D.N., Giardine R. (2023). Committee Opinion Number 432. https://www.acog.org/clinical/clinical-guidance/committee-opinion/articles/2017/03/carrier-screening-for-genetic-conditions.

[23] Vill K., Schwartz O., Blaschek A., Gläser D., Nennstiel U., Wirth B., Burggraf S., Röschinger W., Becker M., Czibere L. (2021). Newborn screening for spinal muscular atrophy in Germany: Clinical results after 2 years. Orphanet. J. Rare Dis..

[24] Schwartz O., Kölbel H., Blaschek A., Gläser D., Burggraf S., Röschinger W., Schara U., Müller-Felber W., Vill K. (2022). Spinal Muscular Atrophy—Is Newborn Screening Too Late for Children with Two SMN2 Copies?. J. Neuromuscul. Dis..

[25] Finkel R.S., Benatar M. (2022). Pre-symptomatic spinal muscular atrophy: A proposed nosology. Brain.

[26] Sumner C.J., Crawford T.O. (2018). Two breakthrough gene-targeted treatments for spinal muscular atrophy: Challenges remain. J. Clin. Investig..

[27] Smith B.A., Trujillo-Priego I.A., Lane C.J., Finley J.M., Horak F.B. (2015). Daily quantity of infant leg movement: Wearable sensor algorithm and relationship to walking onset. Sensors.

[28] Deng W., Vanderbilt D.L., Smith B.A. (2018). Differences in spontaneous leg movement patterns between infants with typical development and infants at risk for developmental delay: Cross-sectional observation prior to sitting onset. J. Mot. Learn. Dev..

[29] Abrishami M.S., Nocera L., Mert M., Trujillo-Priego I.A., Purushotham S., Shahabi C., Smith B.A. (2019). Identification of developmental delay in infants using wearable sensors: Full-day leg movement statistical feature analysis. IEEE J. Transl. Eng. Health Med..

[30] McIntyre M., Dunn L., David J., Devine C., Smith B.A. (2023). Daily Quantity and Kinematic Characteristics of Leg Movement in a Child with SMA (2 Copies SMN2). Pediatr. Phys. Ther..

