# Supplementary material for: A Five-Year Review of Newborn Screening for Spinal Muscular Atrophy in the State of Utah: Lessons Learned
Source: Int J Neonatal Screen. 2024 Jul 22;10(3):54. doi: 10.3390/ijns10030054 (PMC11270276; doi:10.3390/ijns10030054)
Supplement: Supplementary file 1 [file IJNS-10-00054-s001.zip › IJNS-3062509-supplementary.pdf]

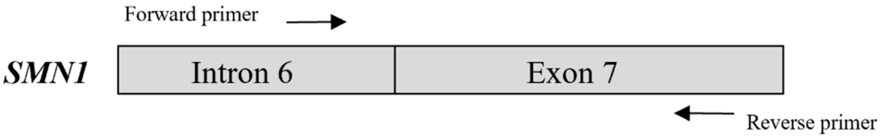

Figure S1. Schematic map indicating the location of qPCR primers used in the Utah newborn screening assay.

Table S1. SMA Newborn Screening Results Interpretation and Follow Up.

Result	Determination	Interpretation	Next step
SMN1 exon 7 **amplified**<br>*RPPH1* **amplified**	Normal	Negative for SMA	No SMA follow-up needed
SMN1 exon 7 **absent**<br>*RPPH1* **amplified**	Abnormal	Positive for SMA	Follow-up with Pediatric Neurology
SMN1 exon 7 **delayed, absent**<br>*RPPH1* **delayed, absent**	Indeterminate	Sample quality concern	Repeat screen
